# New understanding of hardening mechanism of TiN/SiN_*x*_-based nanocomposite films

**DOI:** 10.1186/1556-276X-8-427

**Published:** 2013-10-17

**Authors:** Wei Li, Ping Liu, Yongsheng Zhao, Fengcang Ma, Xinkuan Liu, Xiaohong Chen, Daihua He

**Affiliations:** 1 School of Materials Science and Engineering, University of Shanghai for Science and Technology, Shanghai 200093, China; 2 School of Mechanical Engineering, University of Shanghai for Science and Technology, Shanghai 200093, China

**Keywords:** TiN/SiN_*x *_film, Nanocomposite, Hardening mechanism, Microstructure

## Abstract

In order to clarify the controversies of hardening mechanism for TiN/SiN_*x*_-based nanocomposite films, the microstructure and hardness for TiN/SiN_*x *_and TiAlN/SiN_*x *_nanocomposite films with different Si content were studied. With the increase of Si content, the crystallization degree for two series of films firstly increases and then decreases. The microstructural observations suggest that when SiN_*x *_interfacial phase reaches to a proper thickness, it can be crystallized between adjacent TiN or TiAlN nanocrystallites, which can coordinate misorientations between nanocrystallites and grow coherently with them, resulting in blocking of the dislocation motions and hardening of the film. The microstructure of TiN/SiN_*x*_-based nanocomposite film can be characterized as the nanocomposite structure with TiN-based nanocrystallites surrounded by crystallized SiN_*x *_interfacial phase, which can be denoted by nc-TiN/c-SiN_*x *_model ('c’ before SiN_*x *_means crystallized) and well explain the coexistence between nanocomposite structure and columnar growth structure within the TiN/SiN_*x*_-based film.

## Background

As superhard (hardness *H* ≥ 40 GPa) film material, nanocomposite films have been widely investigated in the past decades for use as wear-resistant coatings on tools and mechanical components [1,2]. Among them, the pseudobinary TiN/SiN_*x *_is a representative film due to strong surface segregation of the constituent phases (TiN and SiN_*x *_have essentially no solid solubility). Especially, since hardness as high as 80 to 105 GPa was reported by Veprek et al. in 2000 [3], it has attracted much attention from the scientific community. So far the nanostructure and hardening mechanism have been widely explained by nc-TiN/a-SiN_*x *_model proposed by Veprek et al. in 1995 [4], in which equiaxed TiN nanocrystallites (nc-TiN) were embedded in an amorphous SiN_*x*_ (a-SiN_*x*_) matrix.

However, this model is in dispute due to the lack of direct experimental evidence, which mainly reflects in two aspects. On one hand, whether TiN crystals are transformed from columnar crystals into equiaxed nanocrystallites is disputed, since there was no direct cross-sectional transmission electron microscopy (TEM) observation for the isotropic nature of the TiN grain. On the other hand, whether SiN_*x *_phase exists as amorphous state is also disputed, since Veprek et al. [4] suggested SiN_*x *_was amorphous because no obvious SiN_*x *_Bragg reflections in X-ray diffraction (XRD) patterns were found, which lacked direct observational evidence so far. Later, based on their high-resolution TEM (HRTEM) observations, Kong et al. [5] reported that TiN were columnar nanocrystals, rather than equiaxed nanocrystals, separated by crystallized SiN_*x *_interfacial phases. Hultman et al. [6] suggested that SiN_*x *_interfacial phase could be crystalline located around TiN nanocrystals according to their *ab initio* calculations. However, they did not give direct experimental evidence. In addition, the cross-sectional TEM published by Zhang et al. [7] and Kauffmann et al. [8] showed that even with increased Si content up to 12 at.%, the TiN/SiN_*x *_nanocomposite films still had a columnar morphology, which increases the uncertainty of the existing model and hardening mechanism of TiN/SiN_*x *_film.

To clarify these controversies about hardening mechanism, TiN/SiN_*x *_and TiAlN/SiN_*x *_nanocomposite films with different Si content were synthesized since the hardness of TiN/SiN_*x*_-based nanocomposite films was highly sensitive to the thickness of SiN_*x *_interfacial phase [3,4]. The relationship between microstructure and hardness for two series of films would be studied. Special attention would be paid to the morphology and structure of constituent phases in two films.

## Methods

### Materials

The TiN/SiN_*x *_and TiAlN/SiN_*x *_nanocomposite films were fabricated on the silicon substrates by reactive magnetron sputtering system. The TiN/SiN_*x *_and TiAlN/SiN_*x *_nanocomposite films were sputtered from TiSi and TiAlSi compound targets (99.99%), respectively, with 75 mm in diameter by RF mode and the power was set at 350 W. The TiSi and TiAlSi compound targets with different Si content were prepared by cutting the Ti (at.%, 99.99%), TiAl (Ti at.%/Al at.% = 70%:30%) and Si targets (at.%, 99.99%), respectively, into 25 pieces and then replacing different pieces of Ti and TiAl with same piece of Si. Adopting this method, TiSi and TiAlSi targets with different Si/Ti (or Si/Ti_0.7_Al_0.3_) volume or area ratios, including 1:24, 2:23, 3:22, 4:21, and 5:20 were prepared. The base pressure was pumped down to 5.0 × 10^-4^ Pa before deposition. The Ar and N_2_ flow rates were 38 and 5 sccm, respectively. The working pressure was 0.4 Pa and substrate was heated up to at 300°C during deposition. To improve the homogeneity of films, the substrate was rotated at a speed of 10 rpm. The thickness of all the TiN/SiN_*x *_and TiAlN/SiN_*x*_ nanocomposite films was about 2 μm.

### Characterization

The microstructures of TiN/SiN_*x *_and TiAlN/SiN_*x *_nanocomposite films were characterized by XRD using a Rigaku D/MAX 2550 VB/PC (Rigaku Corporation, Tokyo, Japan) with Cu Kα radiation and field emission HRTEM using a Philips CM200-FEG (Philips, Amsterdam, Netherlands). The preparation procedures of cross-section specimen for HRTEM observation are as follows: The films with substrate were cut into two pieces and adhered face to face, which subsequently cut at the joint position to make a slice. The slices were thinned by mechanical polishing followed by argon ion milling. The hardness was measured by a MTS G200 nanoindenter (Agilent Technologies, Santa Clara, CA, USA) using the Oliver and Pharr method [9]. The measurements were performed using a Berkovich diamond tip at a load of 5 mN with the strain rate at 0.05/s. The indentation depth was less than one-tenth of the film thickness to minimize the effect of substrate on the measurements. Each hardness value was an average of at least 16 measurements.

## Results and discussion

**Figure 1:**
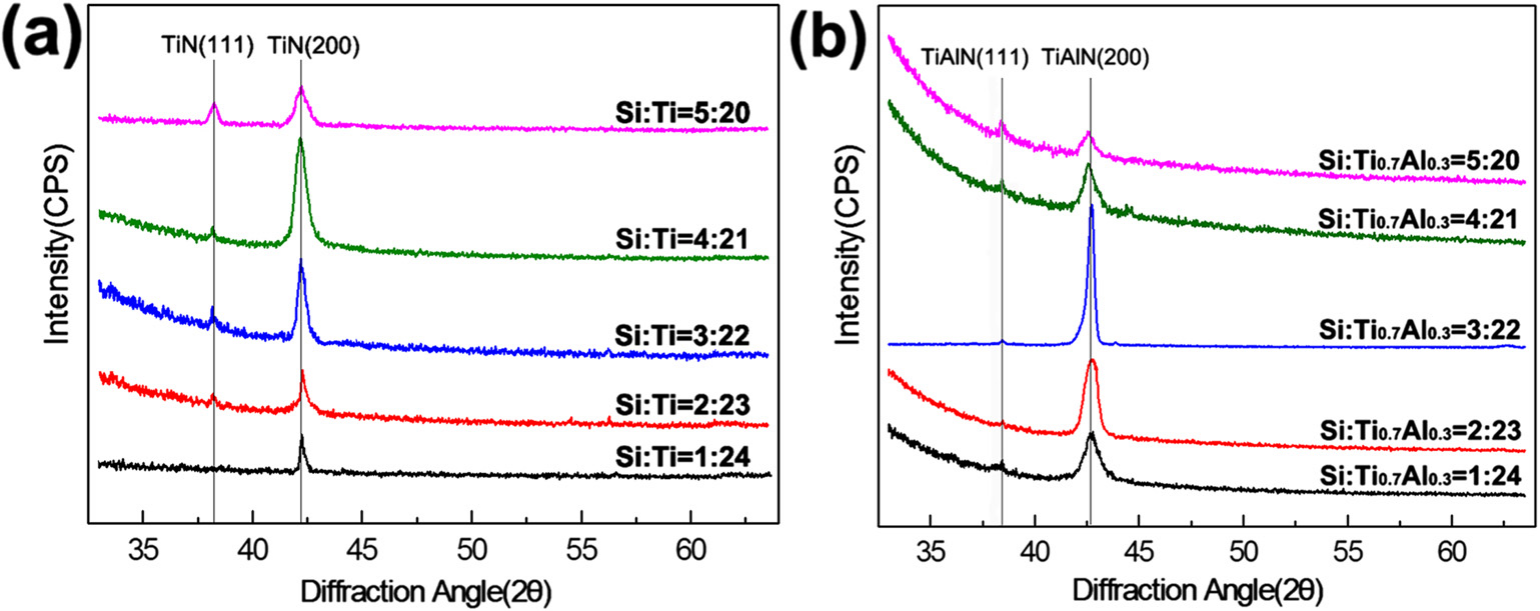
**XRD patterns of (a) TiN/SiN**_***x ***_**and (b) TiAlN/SiN**_***x ***_**nanocomposite films with different Si content.**

The XRD patterns of TiN/SiN_*x *_and TiAlN/SiN_*x*_ nanocomposite films with different Si content are shown in Figure 1. It can be seen that two series of films are only composed of TiN or TiAlN phase, while no SiN_*x*_ phase is detected. Veprek had attributed the absence of SiN_*x*_ phase to its amorphous characteristic [4]. Actually, it can also be explained by low content of SiN_*x*_ phase. Figure 1a,b indicates that TiN/SiN_*x*_ and TiAlN/SiN_*x*_ nanocomposite films both present (200) preferred orientation. With the increase of Si content, the intensities of TiN and TiAlN (200) diffraction peaks firstly increase and then decrease, suggesting that the crystallinity for TiN and TiAlN phases initially improves and then deteriorates. The TiN/SiN_*x*_ and TiAlN/SiN_*x*_ films exhibit the highest crystallinity when Si/Ti (or Si/Ti_0.7_Al_0.3_) ratio is 4:21 and 3:22, respectively.

The influence of Si content on crystallinity throws doubt upon the nc-TiN/a-SiN_*x*_ model proposed by Veprek [3,4]. If SiN_*x*_ phase exists as amorphous state, the increase of Si/Ti ratio from 1:24 to 5:20 (SiN_*x*_ fraction accordingly rises from 4 to 20 at.%) only leads to thickening of amorphous SiN_*x*_ interface, which cannot improve the crystallization degree of film, but lowers it due to the increasing impeditive effect on TiN growth. In addition, as amorphous SiN_*x*_ interfacial phase thickens, TiN and TiAlN phases cannot only present (200) orientation, but may also grow along other directions owing to the randomicity of crystallite growth [10]. Therefore, whether SiN_*x*_ interfacial phase is amorphous deserves to be further deliberated.

In fact, the effect of Si content on crystallinity of TiN/SiN_*x*_ and TiAlN/SiN_*x*_ films brings into our mind the influence of amorphous modulation layer thickness on the crystallization degree of nanomultilayered films, such as TiN/SiC [11], TiAlN/SiO_2_[12], and CrAlN/SiN_*x*_[13]. In these nanomultilayered film systems, with the increase of amorphous layer thickness, the crystallization degree of films firstly increases and then decreases, which can be attributed to two facts. On one hand, the initial increase of amorphous layer thickness could not only crystallize the amorphous layer and grew epitaxially with crystal layer, but also the newly deposited crystal layer could grow epitaxially on crystallized amorphous layer, leading to the 'mutual promotion effect’ of growth in nanomultilayers and improvement of crystallization integrity. The thicker the crystallized amorphous layer thickness is, the higher the crystallization degree of the nanomultilayered film. On the other hand, with further increase of amorphous layer thickness, the amorphous layers cannot keep the crystallization state and change back into the amorphous state, which destructs epitaxial growth structure and decreases the crystallization integrity of the nanomultilayer. Therefore, it can be deduced that with increase of Si content, SiN_*x*_ interfacial phase may undergo the crystallized process, helping to improve the crystallization degree and maintain (200) preferred orientation of TiN/SiN_*x*_ and TiAlN/SiN_*x*_ films. Nevertheless, this deduction needs to be verified from HRTEM observations.

**Figure 2:**
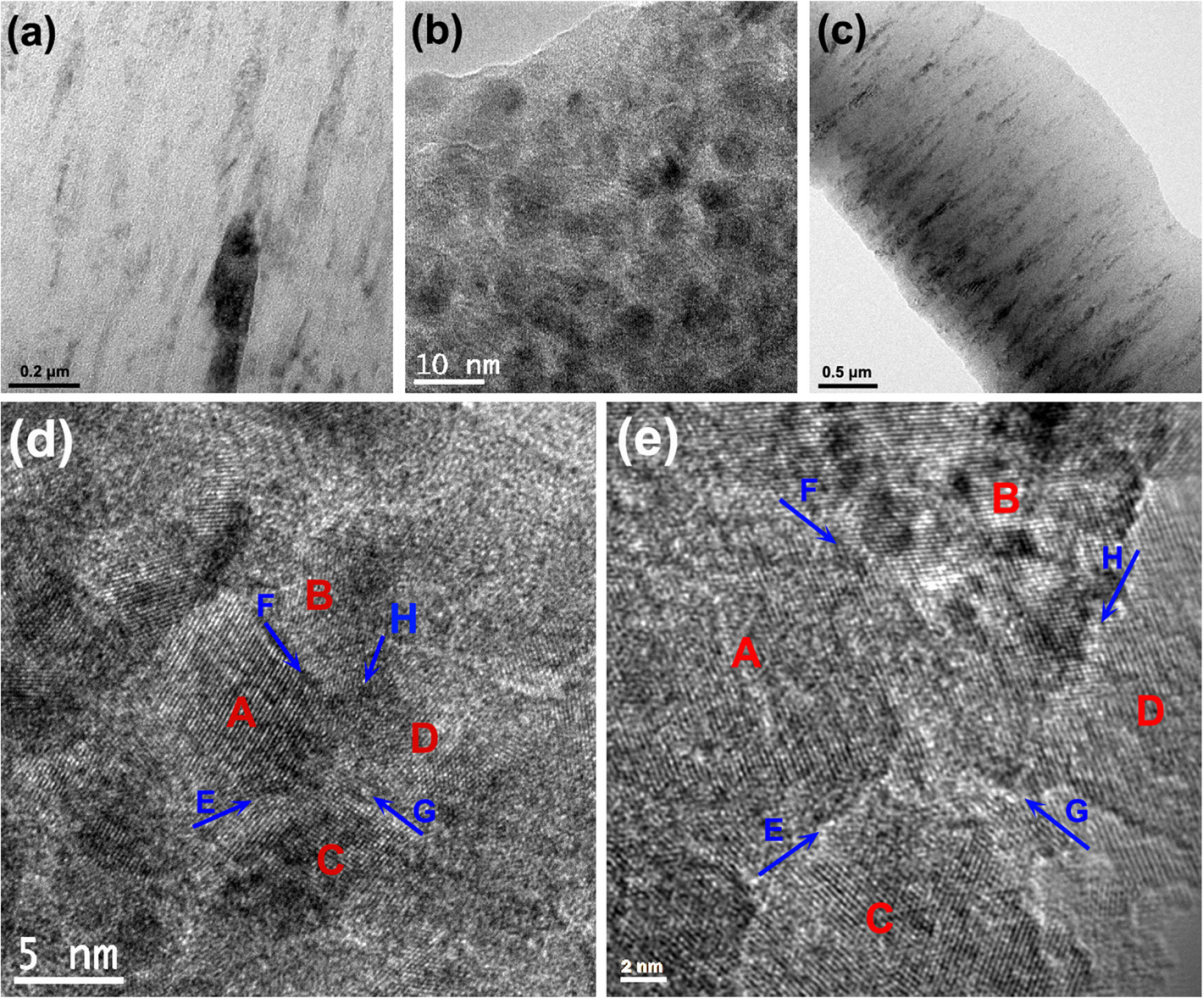
**Cross-sectional HRTEM images of TiN/SiN**_***x ***_**and TiAlN/SiN**_***x ***_**nanocomposite films. (a)** Low magnification, **(b)** medium magnification, **(d)** high magnification for TiN/SiN_*x*_ nanocomposite film (Si/Ti = 4:21), and **(c)** low magnification, **(e)** high magnification for TiAlN/SiN_*x*_ nanocomposite film (Si/Ti_0.7_Al_0.3_ = 3:22). The SiN_*x*_ interfacial phase is observed to exist as crystallized state, rather than amorphous state, such as E zone between A and C crystals, F zone between A and B crystals, H zone between B and D crystals, and G zone between C and D crystals.

Figure 2 presents the typical cross-sectional HRTEM images of TiN/SiN_*x*_ film with Si/Ti ratio of 4:21 and TiAlN/SiN_*x*_ film with Si/Ti_0.7_Al_0.3_ ratio of 3:22. From Figure 2a,c, it is clear that similar with TiN and TiAlN monolithic films, both nanocomposite films show obvious columnar growth structure, in agreement with results from Zhang et al. [7] and Kauffmann et al. [8]. This columnar growth structure cannot be explained by the nc-TiN/a-SiN_*x*_ model. According to the model [4,14], with the addition of Si element, the amorphous SiN_*x*_ interfacial phase interrupted the columnar growth of TiN and divided into many equiaxed TiN nanocrystallites. In this case, the columnar cross-sectional morphology should not be observed, but the amorphous fracture morphology as presented in [15]. However, the columnar cross-sectional structure is obviously observed in both TiN/SiN_*x*_ and TiAlN/SiN_*x*_ films, which brings further question to nc-TiN/a-SiN_*x*_ model.

From Figure 2a, it can also be seen that many small equiaxed nanocrystallites exist within the TiN/SiN_*x*_ film in the cross-sectional morphology. In the medium-magnification image of Figure 2b, it is obvious that many equiaxed nanocrystallites with dark contrast are surrounded by the interfacial phase with bright contrast. According to the nanocomposite structure of TiN/SiN_*x*_ film, it is not difficult to conclude that the equiaxed nanocrystallites with dark contrast and interfacial phase with bright contrast correspond to TiN and SiN_*x*_ phases, respectively, indicating that the film constituted of equiaxed TiN nanocrystallites encapsulated with SiN_*x*_ interfacial phase, rather than columnar-like TiN nanocrystals proposed by Kong et al. [5]. The average size of TiN crystallite is about 6 to 10 nm, with average SiN_*x*_ interfacial thickness of 0.5 to 0.7 nm. In the high-magnification image of Figure 2d, the TiN crystallites basically grow along with the same direction and present the epitaxial growth structure. The SiN_*x*_ interfacial phase is observed to exist as crystallized state, rather than amorphous state, such as the E zone between A and C crystals, F zone between A and B crystals, H zone between B and D crystals, and G zone between C and D crystals, which verifies the validity of above deduction. It is worth noting that there are also some amorphous areas present in Figure 2d. Actually, these regions are not composed of real amorphous phase. When we slightly tilted the specimen, the regions that appeared to be amorphous could change into crystallized structure, which suggested that there existed the misorientation difference between different regions and that the 'amorphous’ regions are not really composed of amorphous phase, but crystallized phase. Therefore, it is reasonably believed that there exists the same 'crystallized effect’ of nanomultilayered films in nanocomposite films, namely, when Si content increases to an appropriate value, that is, SiN_*x*_ interfacial phase reaches to a proper thickness, the SiN_*x*_ interfacial phase can be crystallized under the template effect of adjacent TiN crystallites, which can coordinate the misorientations between TiN crystallites and grow coherently with them. In high magnification of TiAlN/SiN_*x*_ film, it can also be observed that the lattice fringes continuously go across adjacent TiAlN crystallites through SiN_*x*_ interfaces, suggesting that SiN_*x*_ phase has been crystallized between adjacent TiAlN crystallites and grows coherently with them (Figure 2e). Comparatively, the SiN_*x*_ interfacial thickness of TiAlN/SiN_*x*_ film is smaller (about 0.3 to 0.5 nm) based on Figure 2e than that (about 0.5 to 0.7 nm) of TiN/SiN_*x*_ film in Figure 2d, which is agreement with the fact that the Si/Ti_0.7_Al_0.3_ ratio of 3:22 in TiAlN/SiN_*x*_ film is lower than Si/Ti ratio of 4:21 in TiN/SiN_*x*_ film.

**Figure 3:**
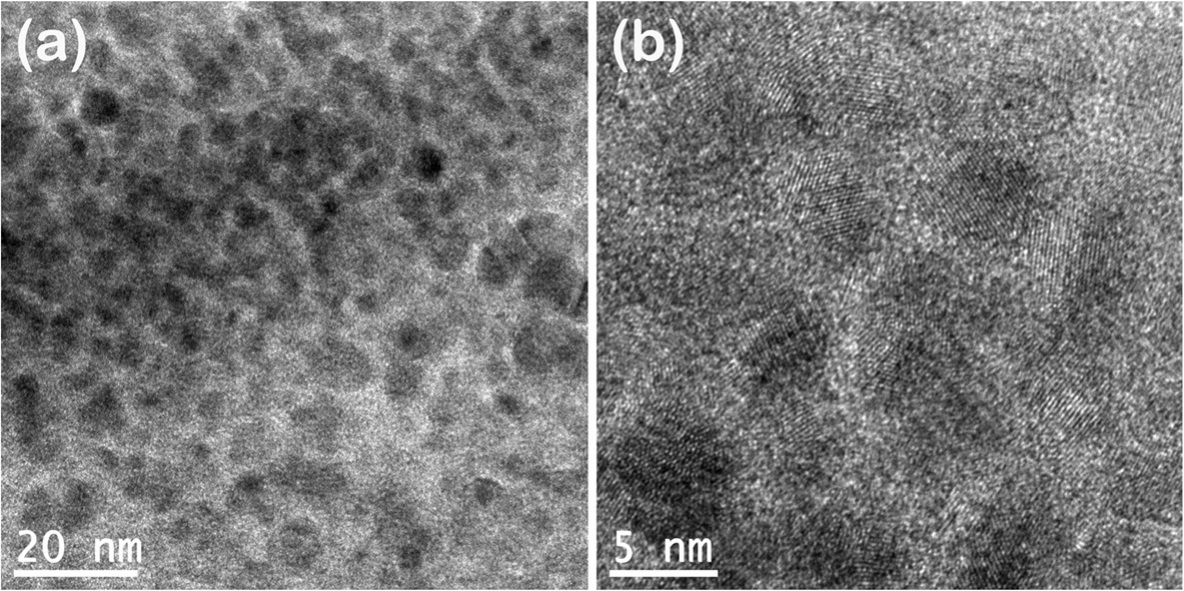
**Cross-sectional HRTEM images of TiN/SiN**_***x ***_**nanocomposite film with high Si content (Si/Ti = 5:20). (a)** Low magnification and **(b)** high magnification.

Figure 3 shows that the typical cross-sectional HRTEM images of TiN/SiN_*x*_ nanocomposite film with Si/Ti ratio of 5:20. It can be seen from Figure 3a that the thickness of SiN_*x*_ interfacial phase increases compared with Figure 2b (Si/Ti = 4:21). The average size of TiN crystallite is about 4 to 8 nm, smaller than that in Figure 2b (6 to 10 nm). From the high-magnification image in Figure 3b, it can be seen that SiN_*x*_ interfacial phase presents amorphous state, rather than crystallized state, suggesting that SiN_*x*_ interfacial phase cannot maintain the crystallization with high interfacial phase thickness and transforms back into amorphous state. Amorphous SiN_*x*_ interfacial phase breaks the epitaxial growth structure between the adjacent TiN nanocrystallites, leading to the various growth misorientations for TiN nanocrystallites, as shown in Figure 3b.

**Figure 4:**
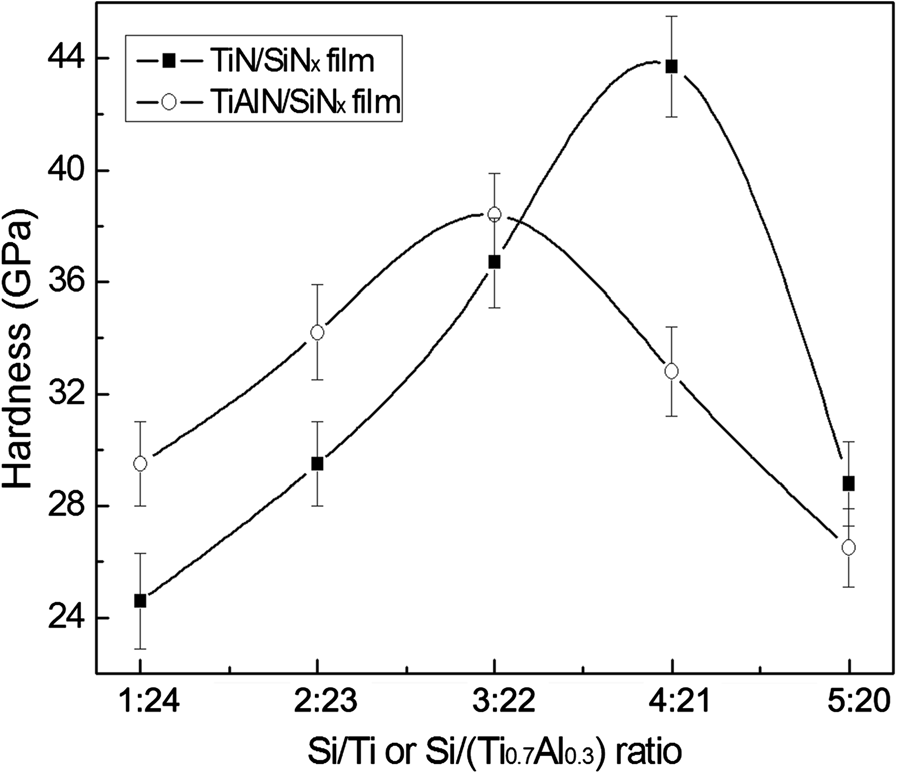
**Variation of hardness of TiN/SiN**_***x ***_**and TiAlN/SiN**_***x ***_**nanocomposite films with change of Si content.**

According to the above analysis, TiN/SiN_*x*_ and TiAlN/SiN_*x*_ nanocomposite films have the same interfacial morphological evolution with nanomultilayered films. If this is a fact, the TiN/SiN_x_ and TiAlN/SiN_x_ nanocomposite films should be effectively strengthened when SiN_*x*_ interfacial phase is well crystallized and the film presents the highest crystallization degree. Figure 4 shows the dependence of hardness for two films on Si content. It can be seen that the hardness values for two films both firstly increase and then decrease with increase of Si content. TiN/SiN_*x*_ and TiAlN/SiN_*x*_ films achieve the maximal hardness values of 43.7 and 38.4 GPa, respectively, with Si/Ti (or Si/Ti_0.7_Al_0.3_) ratio of 4:21 and 3:22, which validates our deduction.

It is not difficult to find that the variation of hardness with increase of Si content is in accord with crystallization degree. According to the hardening mechanism proposed in nc-TiN/a-SiN_*x*_ model [3,4,14], the TiN crystallite size is too small for dislocation activities, and the film can only deform by grain boundary sliding (i.e., by moving single undeformed TiN nanocrystallites against each other). However, based on this mechanism, TiN nanocrystallites that slide along grain boundary must cause the coordinate movement of adjacent nanocrystallites, such as crystallite rotation and shift [16], and leave trace in the sliding boundary, which both lack direct experimental evidence from the existing literatures. In addition, the dependence of hardness on Si content should not have related to crystallization degree.

**Figure 5:**
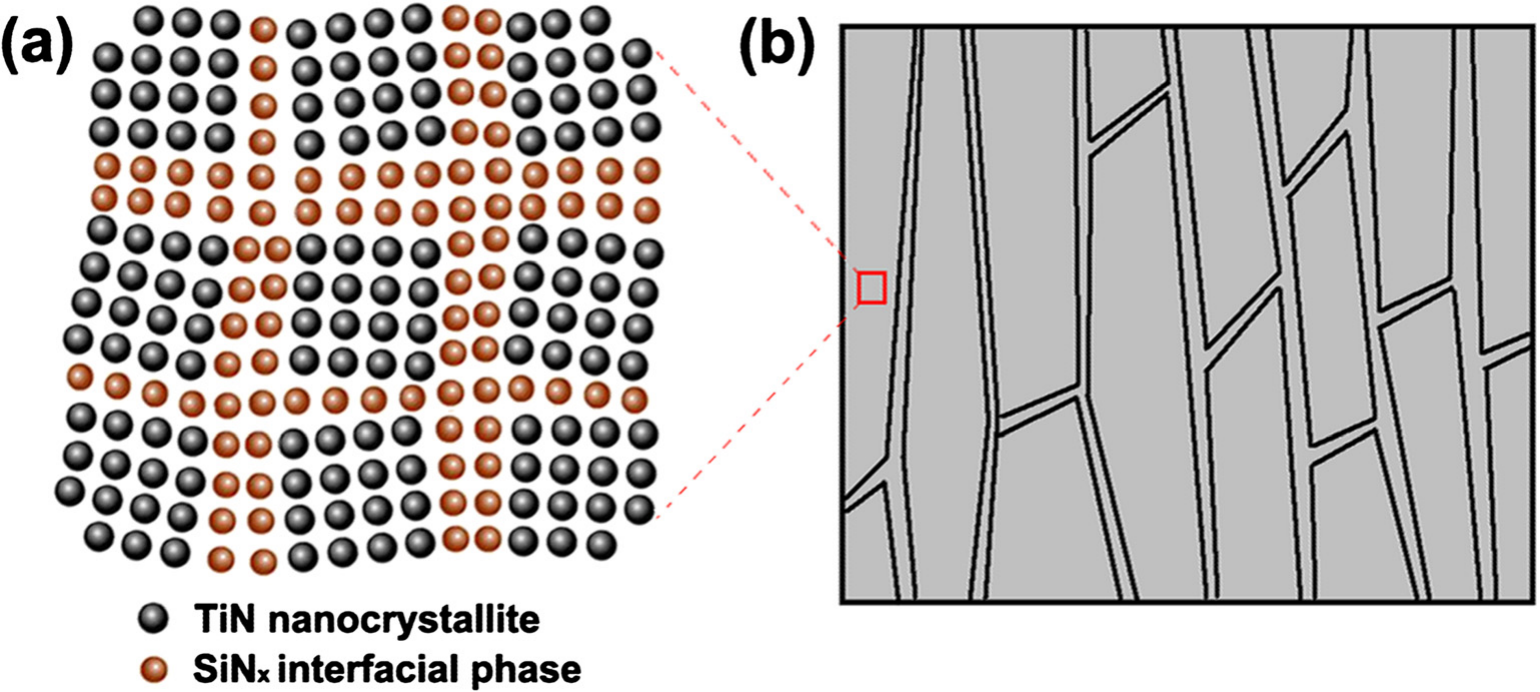
**Cross-sectional schematic diagrams. (a)** Nanoscale configuration (nc**-**TiN**/**c**-**SiN_*x*_ model) and **(b)** columnar crystals within TiN/SiN_*x*_ nanocomposite film (the red frame and the dash line show that **(a)** is the microstructural model of the local zone within **(b)**).

Actually, we believe that with the initial increase of Si content, SiN_*x*_ interfacial phase with low thickness inclines to grow epitaxially on the surface of TiN nanocrystallites in order to lower the interfacial energy between TiN and SiN_*x *_[17]. When the newly arriving TiN deposits on SiN_*x*_ surface, it inclines to grow along the original direction. As a result, SiN_*x*_ interfacial phases present to be crystallized, transferring the growth direction and maintaining the epitaxial growth structure between the adjacent TiN nanocrystallites, as shown in the schematic diagram of Figure 5a. In this case, the nanocomposite film can exhibit the characteristic of nanomultilayered films in the local area, as shown in Figure 5a. According to Koehler's modulus difference strengthening theory [18], when the dislocations traverse across the coherent interface in nanomultilayer, the dislocation motions are hindered at interface by the force that is generated from the two layers with different shear moduli, which can effectively strengthen the film. Furthermore, the compressive and tensile stress fields are created at the coherent interface due to the difference of lattice parameter between two layers, which can also block the movement of dislocations and be partially responsible for the hardening effect [19]. It is worth noting that due to the low crystallization degree at low Si content, the epitaxial growth structure is not well formed. Therefore, the impeding effect of coherent interface on dislocation motion decreases, resulting in the comparatively low hardness of film with low Si (Si/Ti ratio is below 4:21 or Si/Ti_0.7_Al_0.3_ ratio is below 3:22). The epitaxial growth between TiN or TiAlN and SiN_*x*_ cannot disturb the columnar growth structure due to the improvement effect on crystallization degree, but can exist inside the columnar crystals of TiN/SiN_*x*_ and TiAlN/SiN_*x*_ films, as illustrated in Figure 5b; namely, the nanocomposite structure with TiN nanocrystallites surrounded by crystallized SiN_*x*_ interfacial phase (expressed as nc-TiN/c-SiN_*x*_ model, 'c’ before SiN_*x*_ means crystallized) and columnar growth structure can coexist within the TiN/SiN_*x*_ film, which is a typical difference from former nc-TiN/a-SiN_*x*_ model.

Nevertheless, with further increase of Si content, the SiN_x_ interfacial phase thickens and cannot maintain the crystallized state between adjacent TiN nanocrystallites, resulting in the transformation back into the amorphous state and breakage of epitaxial growth structure. Accordingly, the blocking effects on the dislocation motions decrease. Despite that the amorphous phase can also act as an obstacle for dislocation movement, its impeding effect on the dislocation motion is much smaller than that of coherent interface. Therefore, the hardness of the film decreases. It is worth noting that the Si/Ti ratio at which film presents the highest crystallinity and hardness for TiAlN/SiN_*x*_ film is 3:22, lower than that of 4:22 for TiN/SiN_*x*_ film. That is to say, the maximal crystallized SiN_*x*_ interfacial thickness maintained by TiAlN is smaller than that by TiN, which can be attributed to the misfit difference between TiN/SiN_*x*_ and TiAlN/SiN_*x *_[14]. The lattice parameter of TiN decreases with the addition of Al [20], resulting in the increase of misfit between TiAlN and SiN_*x*_, which reduces the epitaxial breakdown thickness of SiN_*x*_ and might also be the reason for lower maximal hardness for TiAlN/SiN_*x*_ film relative to TiN/SiN_*x*_ film.

## Conclusions

In summary, in order to clarify the controversies of hardening mechanism for TiN/SiN_*x*_-based nanocomposite films, the microstructure and hardness for TiN/SiN_*x*_ and TiAlN/SiN_*x*_ nanocomposite films with different Si content were studied. With the increase of Si content, the crystallization degree for two series of films firstly increases and then decreases. The microstructural observations suggest that when SiN_*x*_ interfacial phase reaches to a proper thickness, it can be crystallized between adjacent TiN or TiAlN nanocrystallites, which can coordinate misorientations between nanocrystallites and grow coherently with them, resulting in blocking of the dislocation motions and hardening of the film. The microstructure of TiN/SiN_*x*_-based nanocomposite film can be characterized as the nanocomposite structure with TiN-based nanocrystallites surrounded by crystallized SiN_*x*_ interfacial phase, which can be denoted by nc-TiN/c-SiN_*x*_ model ('c’ before SiN_*x*_ means crystallized) that can well explain the coexistence between the nanocomposite structure and columnar growth structure within the TiN/SiN_*x*_-based film.

## Competing interests

The authors declare that they have no competing interests.

## Authors' contributions

WL designed the experiment and wrote the article. PL, YZ, and FM carried out the synthesis of TiN/SiN_*x*_ and TiAlN/SiN_*x*_ nanocomposite films. XL, XC, and DH assisted in the technical support for measurements (XRD, HRTEM, and nanoindention) as well as the data analysis. All authors read and approved the final manuscript.
